# Employees and the National Insurance Act

**Published:** 1912-09-21

**Authors:** 


### NATIONAL INSURANCE ACT DEPARTMENT.

### EMPLOYEES AND THE NATIONAL INSURANCE ACT.

### XXVIII.—The Contract of Membership of Approved Societies.

A very important Memorandum, numbered 119, dealing with the membership of approved societies, was issued by the Insurance Commissioners on September 5. Some amount of attention has already been paid to this Memorandum in the public press by reason of the fact that it aroused adverse comment at the recent Trade Unions Congress. Unfortunately, the meaning of the Memorandum had apparently been misunderstood by those who spoke against it, and in order to prevent any similar misunderstanding on the part of our readers we propose to give a description of its contents. Before doing so, however, it is necessary to make a few preliminary remarks. In the first place, it has to be borne in mind that no person can be a member of more than one society for the purpose of obtaining the benefits of the National Insurance. Any infringement of this rule would be an offence against the Act, and would be punishable accordingly. It therefore follows that no two societies can claim the same person as a member, and this is how the difficulty has arisen. The free choice of a society is the one piece of liberty allowed to the insured person under the Act, and any attempt at coercion on the part of employers or others to obtain members for a particular society is not allowed. At the same time the various approved societies have been pressing their claims upon likely members, and by peaceful persuasion have secured large numbers of applications from persons who have previously signed similar forms for other societies. Each society has considered such a person as one of its members, and having gone to the trouble of issuing an insurance card and book, and registered the person in the membership register, each society has looked forward to receiving the duly stamped card at the end of the first quarter—in the middle of next month—in order to be able to claim credit from the Commissioners on account of such person, of the initial reserve and the quarter's contributions, out of which, by the way, the expenses incurred in enrolling members are to be met.

#### The Scramble for Cards.

The difficulty was foreshadowed even before July 15 last, by which time there was considerable ground for apprehension that unless stringent measures were taken there would be an unseemly scramble for cards; the representative of each society endeavouring to collect not only the cards that had been issued by his own society, but also those that had been issued by rival organisations. It was imperative that there should be no such scramble for cards, and it was the main object of the Memorandum just issued to prevent any such ocurrence by means of special arrangements as to the transfer of members from one society to another at the end of the first quarter.

The second important point that must be kept in view is that by a strict adherence to the provisions of the Act a society is not obliged to consent to the transfer of a member to another society, and if it can show that its consent has not been unreasonably withheld it will not have to part with the transfer value attaching to such member. The question of what was to be considered as the determining factor in fixing the membership with a particular society was, however, the crux of the situation. This is not prescribed in the Act, neither had any ruling been given in the matter by the Commissioners. Two opposite views had been taken by the societies. On the one hand, it was claimed that, as the choice of a society need not be made until the time for sending in the first card, the society that obtained the card also obtained the member named on that card, no matter how many applications had been made by such persons to other societies, and whether they had been accepted or not. This is the view that would have lead to the scramble for cards, and the one that has been adopted by society officials who were late in making their arrangements, or who only awoke to the importance of securing members when others had already been hard at work. The other view was that membership of a society was completed when an application for admittance as a member had been accepted by the society, either expressly or by the issue to such applicant of the insurance card or book, or of the card and book. This is the view that has been taken of the matter by the Insurance Commissioners, and there are good grounds on which it can be supported. Insurance is essentially a matter of contract. The contract is completed when the acceptance of the risk by the insurer is evidenced, and the evidence of the acceptance of the risk in the case of the National Insurance is made sufficiently clear by the issue of the contribution card or the insurance book, which latter in all cases bears the name of the society and the membership number in the society of the person to whom it is issued. A society which has admitted a member in this way has the right of claiming the reserve applicable to the case even though it may be impossible for it to be ever called upon to make any payment by way of benefits, as for instance in the case which recently came to our notice of a man who, having joined a society and was at work on July 15, died within the first week of the Act coming into operation.

#### The Commissioners' Memorandum.

The Memorandum is entitled "Special Arrangements in regard to Change of Membership during the first six months and the Claiming of Reserve and Transfer Values in such cases." The scheme is really in the nature of a compromise, and its object, as pointed out in a covering letter from the Secretary to the Joint Committee, is to meet the case of those persons who joined a society without giving the matter much thought, and who, on further consideration, decide that another society is better suited to their requirements. The Commissioners express the opinion that it would not be reasonable nor politic for societies to seek to retain such members against their inclination. From this it would seem to follow that, in the Commissioners' opinion, there are no grounds at the present time on which a society could support the contention that its consent to transfer had not been unreasonably withheld. It is pointed out, on the other hand, that admittance into membership involves obligations on the member, and no society can properly admit a person who has already joined another society without being satisfied that the membership in the first society has been cancelled by a regular withdrawal.

### The Use of Withdrawal Forms.

Some few societies had withdrawal forms printed even before July 15, and these were brought into use in cases where the person successfully canvassed had previously been admitted a member of another society. Doubt had been expressed in some quarters as to the validity of these forms, but it would now appear that their use has been definitely recognised and sanctioned. Much of the detail of the Memorandum is only of interest to society officials and committees of management, as it deals with the manner in which the withdrawal notices are to be collected, recorded, and delivered to the societies from which transfers are being made. Apart from these details the general lines of the scheme are of interest to the members, and thus to a large number of our readers, because the welfare of their societies may be involved as well as their own membership.

Briefly explained, the plan is for the society which is claiming a member (Society B) to obtain from that member a signed notice of withdrawal from the society previously joined (Society A). Society B will then send the withdrawal notices of such members as it desires to receive from Society A to each such society, and will itself keep a list of the notices that have been delivered. Then, instead of Society A collecting the insurance cards from the insured persons at the end of the quarter, Society B will carry out this operation, and will at the same time deliver the cards for the second quarter. The cards for the first quarter so collected are then to be marked with the word "Transferred" and sent with the insurance books to Society A. Society A will then make a claim on the Commissioners for the initial reserve value in the same way as for cases of continuing members, and Society B will in like manner claim from them the transfer value. The net result of these operations is to be that, in the event of the notice of withdrawal having been delivered to Society A by September 30, it will receive, on balance, the allowance of 1s. per member to cover the initial expenses incurred in setting the society in operation, together with a further 5d. out of the 11d. allowed for management expenses for the first quarter. If the notices of withdrawal are not delivered until after September 30 Society A is to obtain the benefit of the full allowance of 11d. for management expenses as well as the 1s. for preliminary expenses, together with a further sum of 5d. at the end of the second quarter for the expenses of that quarter.

Society B will, under the arrangement, obtain the balance of 6d. from the management expenses allowed for the quarter as at the end of which the transfer is to date.

It is to be noted, however, that these arrangements are not binding on the societies. They had the option of accepting or of rejecting them, and they were allowed until September 18 to decide whether to adopt them or not. It is also important to note that the arrangements do not hold if withdrawal was effectively notified before July 15, in which case Society B would obtain the full reserve member without the necessity of claiming the transfer from Society A.

In our opinion these suggestions of the Commissioners show a keen desire to make the Act work as smoothly as possible, and they certainly appear to deal fairly with conflicting interests in a way that should be acceptable to all parties. It is a commonplace to say that a compromise never suits either party, but it is clear that without some scheme, under which it will be necessary to give as well as take, the harmonious working of the societies will be in serious jeopardy, and it is our hope that the suggestions will be adopted in the spirit in which they have so manifestly been drafted.

### Acceptance by Trade Unions Officials.

As we go to press we learn that the principal leaders of the Trade Unions have accepted the Commissioners' proposals. This acceptance was signified at a meeting of the Parliamentary Committee of the Trade Unions Congress held jointly with the Management Committee of the General Federation of Trade Unions Approved Societies at which a representative of the Home Office explained the position in detail. The resolution, which was signed by the members attending the meeting, advised the Trade Unions Approved Societies to agree to the terms of the Memorandum, and that the signing of withdrawal forms should immediately be proceeded with. The matter was to be finally decided at a special meeting of the Unions on September 18.

Thus there are grounds for hoping that the fears which were at first entertained as to the success of the scheme have been allayed.

### Answers to Correspondents.

D. B., Essex, informs us that she is a matron of a home managed by a committee, and asks our advice as to her position. As an employed person it would certainly seem that she must be insured, and, being a trained nurse, we advise her immediately to apply to the Secretary of the Nurses' Insurance Society, 15 Buckingham Street, Strand, London, W.C., for full particulars to enable her to apply for membership before October 14. The nurse temporarily employed at the home should also be insured.

NOTE.—Questions and Correspondence on all doubtful points are invited, and should be addressed to the Editor, The Hospital, 28 Southampton Street, Strand, W.C., and marked "Insurance."

